# Sertoli cell-enriched proteins in mouse and human testicular interstitial fluid

**DOI:** 10.1371/journal.pone.0290846

**Published:** 2023-09-01

**Authors:** Liza O’Donnell, Laura F. Dagley, Michael Curley, Annalucia Darbey, Peter J. O’Shaughnessy, Thorsten Diemer, Adrian Pilatz, Daniela Fietz, Peter G. Stanton, Lee B. Smith, Diane Rebourcet

**Affiliations:** 1 Griffith University, Parklands Drive, Southport, Queensland, Australia; 2 Centre for Reproductive Health, Hudson Institute of Medical Research, Clayton, Victoria, Australia; 3 Monash University, Clayton, Victoria, Australia; 4 Department of Medical Biology, Walter and Eliza Hall Institute, University of Melbourne, Parkville, Victoria, Australia; 5 MRC Centre for Reproductive Health, University of Edinburgh, The Queen’s Medical Research Institute, Little France Crescent, Edinburgh, United Kingdom; 6 College of Engineering, Science and Environment, The University of Newcastle, Callaghan, NSW, Australia; 7 School of Biodiversity, One Health & Veterinary Medicine, College of Medical, Veterinary and Life Sciences, University of Glasgow, Garscube Campus, Glasgow, United Kingdom; 8 Medical Faculty, Department of Urology, Pediatric Urology and Andrology, Justus-Liebig-University Giessen, Giessen, Germany; 9 Institute for Veterinary Anatomy, Histology and Embryology, Justus-Liebig-University Giessen, Giessen, Germany; National Institute of Child Health and Human Development (NICHD), NIH, UNITED STATES

## Abstract

Sertoli cells support the development of sperm and the function of various somatic cells in the interstitium between the tubules. Sertoli cells regulate the function of the testicular vasculature and the development and function of the Leydig cells that produce testosterone for fertility and virility. However, the Sertoli cell-derived factors that regulate these cells are largely unknown. To define potential mechanisms by which Sertoli cells could support testicular somatic cell function, we aimed to identify Sertoli cell-enriched proteins in the testicular interstitial fluid (TIF) between the tubules. We previously resolved the proteome of TIF in mice and humans and have shown it to be a rich source of seminiferous tubule-derived proteins. In the current study, we designed bioinformatic strategies to interrogate relevant proteomic and genomic datasets to identify Sertoli cell-enriched proteins in mouse and human TIF. We analysed proteins in mouse TIF that were significantly reduced after one week of acute Sertoli cell ablation *in vivo* and validated which of these are likely to arise primarily from Sertoli cells based on relevant mouse testis RNASeq datasets. We used a different, but complementary, approach to identify Sertoli cell-enriched proteins in human TIF, taking advantage of high-quality human testis genomic, proteomic and immunohistochemical datasets. We identified a total of 47 and 40 Sertoli cell-enriched proteins in mouse and human TIF, respectively, including 15 proteins that are conserved in both species. Proteins with potential roles in angiogenesis, the regulation of Leydig cells or steroidogenesis, and immune cell regulation were identified. The data suggests that some of these proteins are secreted, but that Sertoli cells also deposit specific proteins into TIF via the release of extracellular vesicles. In conclusion, we have identified novel Sertoli cell-enriched proteins in TIF that are candidates for regulating somatic cell-cell communication and testis function.

## Introduction

Sertoli cells are the orchestrator of testis differentiation and are crucial to the support of somatic and germ cells. They are multi-functional cells that coordinate the development of immature spermatogonia into mature elongated spermatids that are released from the tubules at the end of spermatogenesis. The highly dynamic Sertoli cells simultaneously support up to five different types of germ cells, in various phases of development, and provide key signals and structural and nutritional support to guide germ cell survival, mitosis, meiosis and differentiation [1,2].

Sertoli cells also support the function of other testicular cells. They direct the development of peritubular myoid cells that surround the seminiferous tubules and support the function of the testicular vasculature [3–6]. Sertoli cells influence the phenotype and function of interstitial immune cells, modulating testicular homeostasis and responses to infection and inflammation [7]. Sertoli cells also regulate the development and function of the steroidogenic Leydig cells [4–6]. The number of Sertoli cells in the mouse testis during early postnatal development dictates the size of the Leydig cell population in adulthood [8]. Ablation of adult Sertoli cells, but not germ cells, reduces adult Leydig cell number by 75% due to the induction of apoptosis [1,4,9]. The fact that adult Leydig cell number is dependent on the number of Sertoli cells formed during development [8] suggests Sertoli cells produce paracrine factors that support the development of the Leydig cell population. Ablation of adult Sertoli cells also significantly reduces the amount of circulating testosterone after hCG stimulation [3]. Although Sertoli cell-derived paracrine factors are known to regulate adult Leydig cell steroidogenesis [10,11], the specific factors involved are not well understood. Sertoli cell *Wt1* expression and *Wt1*-dependent production of desert hedgehog (DHH) are likely candidates [12]. Defining the factors released by Sertoli cells that maintain somatic cell function could provide new opportunities to design therapies to support different testicular cell functions. Supporting Leydig cell steroidogenesis is particularly important because they produce testosterone that is essential for spermatogenesis and for virility of the male.

The interstitial space between the seminiferous tubules, where the Leydig cells, immune cells and the vasculature are, is bathed in a protein-rich fluid called testicular interstitial fluid (TIF). TIF is a rich source of proteins involved in cell-cell communication in the testis. We recently identified and quantified thousands of proteins in TIF from adult mice and men [13]. Quantitation of the TIF proteome from mice with acute ablation of adult Sertoli cells from the seminiferous epithelium revealed a significant decrease in approximately one third of TIF proteins, indicating that the seminiferous tubules contribute many proteins to TIF [13]. This previous study reported on the surprising finding that many germ cell-derived, and spermatid-specific, proteins are deposited by Sertoli cells into TIF [13]. However, proteins that are produced by Sertoli cells were not assessed.

In current study we aimed to identify Sertoli cell-enriched proteins in TIF from mice and men. We designed a bioinformatic strategy to identify Sertoli cell-enriched proteins in TIF utilising data from the mouse and human TIF proteome [13], previously published testis cell datasets [14,15] and online knowledge databases including the Human Protein Atlas [16]. The results identify Sertoli cell-derived proteins that are candidates for mediating paracrine interactions between the seminiferous tubules and the interstitial cells of the testis and that could be further investigated for a functional role in regulating Leydig cells, resident immune cells and/or the vasculature.

## Materials and methods

### Ethics approvals

Mice were housed and bred under standard conditions of care. Experiments were conducted with licenced permission under the UK Animal Scientific Procedures Act (1986), Home Office licence number PPL 60/4200. All human procedures performed were in accordance with the ethical standards of the Institutional and/or National Research Committee and with the 1964 Helsinki Declaration and its later amendments or comparable ethical standards. All patients were counselled preoperatively and gave written informed consent to perform testicular surgery and collect TIF. This study was approved by the local institutional review board in 2011 and patients for TIF collection were recruited from May 2012 onwards (Ethik-Kommission am FB 11 “Humanmedizin”, Justus-Liebig-Universität Giessen; Ref. No. 26/11).

### Assessment of the TIF proteome from normal mice and mice with acute Sertoli cell ablation

The mouse TIF proteome from phenotypically normal (control) mice and those with Sertoli cell ablation has been previously published [13]. Briefly, transgenic adult male mice (>70 days) expressing diptheria toxin receptor (DTR) specifically in testicular Sertoli cells (Amh-Cre+/+;iDTR+/+ mice) were injected with a single dose of 100ng diptheria toxin (DTX) to ablate the Sertoli cells (DTX group, n = 11). Control mice received vehicle (control group, n = 12) [4]. Mice were culled 1 week later using CO_2_ asphyxiation and cervical dislocation, and testes and TIF were collected as previously described [13,17].

TIF protein concentrations were determined by the BCA method (Pierce, Rockford). Equal amounts of mouse TIF lysate (60 μg) from DTX (n = 11) and control (vehicle-treated) mice (n = 12) were prepared for mass spectrometry analysis as described previously [13]. Mouse TIF peptides were separated and identified using a nano-flow HPLC (M-class, Waters) coupled to an Impact II UHR-QqTOF mass spectrometer (Bruker, Bremen, Germany) [13]. Raw files consisting of high-resolution MS/MS spectra were processed with MaxQuant (version 1.5.8.3) for feature detection and protein identification using the Andromeda search engine and extracted peak lists were searched against the UniProtKB/Swiss-Prot Mus musculus database [13]. Statistically-significant changes in protein expression between the DTX and control mouse TIF samples (adjusted p value <0.05) were identified using the default workflow in the R package Proteus (version 0.2.10) where quantitation was performed at the peptide level [13].

The lists of proteins identified in adult normal mouse TIF (n = 3902), and those that were quantitively compared in TIF from normal (control) vs mice with Sertoli cell ablation (DTX) (n = 3551) were downloaded as Excel files from our previous study [13]. This dataset has been previously used to identify germ cell-enriched proteins in mouse TIF but has not been investigated for Sertoli cell-enriched proteins.

### Assessment of the human TIF proteome

Human TIF samples were obtained from three men with a phenotype of obstructive azoospermia, where sperm are prevented from entering the ejaculate due to a distal obstruction. Spermatogenesis in the testis is usually normal in these patients [18]. The 3 patients used in this study had clinical data indicative of normal testicular function and clinical analysis of testis biopsies by trained staff revealed a normal phenotype [13]. TIF was collected by experienced microsurgeons from patients undergoing M‐TESE (microsurgical‐assisted testicular sperm extraction) for sperm retrieval, as described [13,19]. Prior to dissection of the seminiferous tubules for sperm retrieval, TIF was recovered adjacent to the tubules by applying gentle pressure on the tissue. An average of 200‐500 μL TIF was collected per testis and immediately snap‐frozen in Eppendorf tubes over dry ice in the operating theater and subsequently stored at −80° [13].

For each patient, 200 μg of protein was prepared for mass spectrometry analysis using the USP3 protocol as previously described [13]. Human TIF required prefractionation to resolve the in-depth proteome and thus tryptic peptides from each of the 3 human TIF samples were subjected to high pH reversed phase analysis as previously described [13]. The proteome of human TIF was resolved by mass spectrometry as per mouse TIF, and peptides were searched against the UniProtKB/Swiss-Prot homo sapiens database as previously described [13]. The list of proteins identified (n = 4720) in the 3 human TIF samples was downloaded as an Excel file from our previous publication [13]. This dataset has previously been used to identify germ cell-enriched proteins in human TIF [13] but has not been investigated for Sertoli cell-enriched proteins.

### Identification of Sertoli cell-enriched proteins in mouse TIF

**Fig 1:**
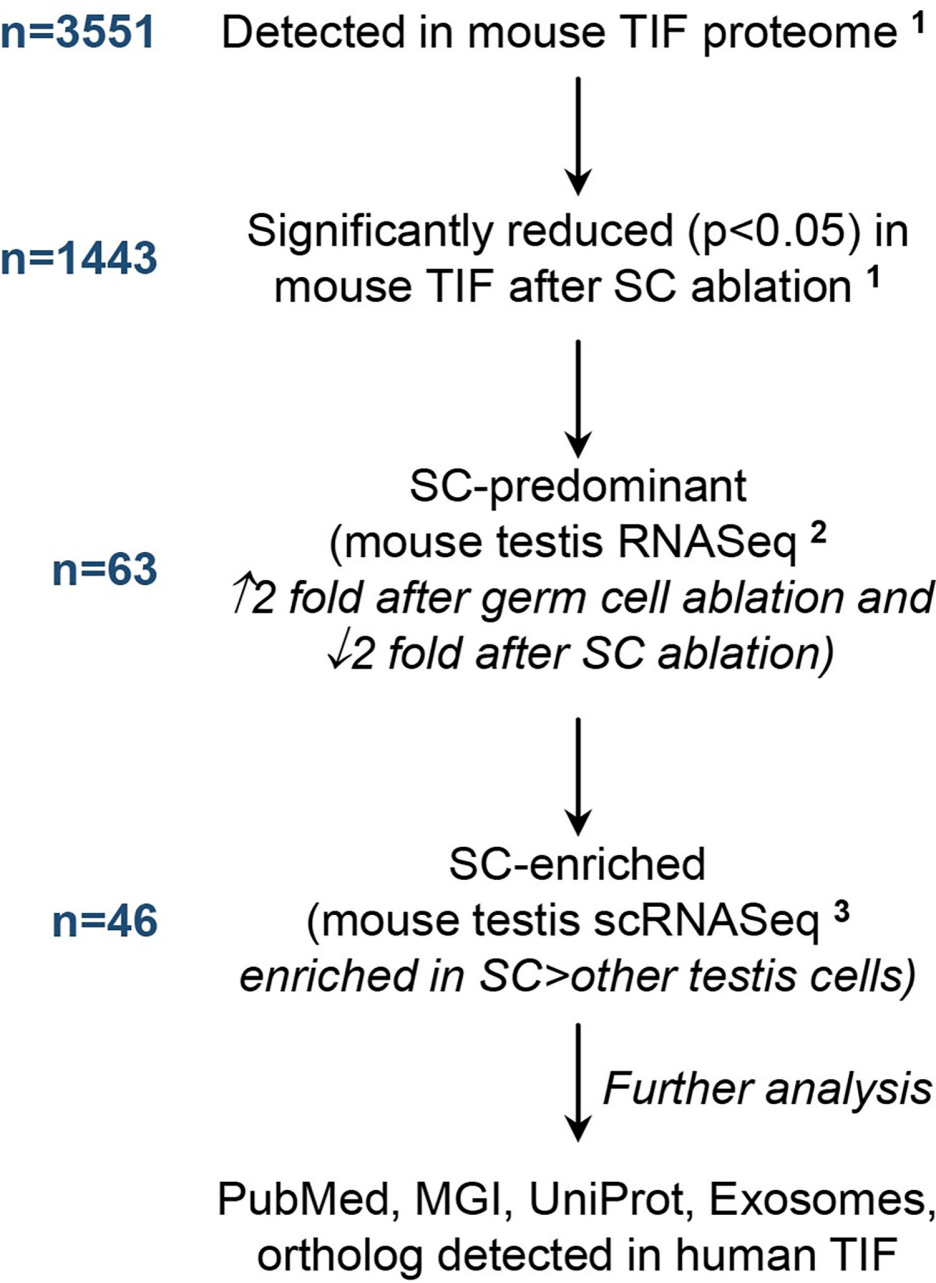
Bioinformatic analysis used to identify Sertoli cell-enriched proteins in mouse testicular interstitial fluid. *n* indicates the number of proteins that were identified at each step in the analysis. SC = Sertoli cells, MGI = Mouse Genome Informatics. ^1^ data from [13], ^2^ data from [15], ^3^ data from [14].

We utilised several independently generated datasets to identify Sertoli cell-enriched proteins in mouse TIF (Fig 1). First, we utilised the adult mouse TIF proteome from control adult mice (n = 12) and mice in which adult Sertoli cells had been acutely ablated for 1 week (n = 11) in a well characterised model involving the administration of diptheria toxin (DTX) to transgenic mice expressing DTX receptor in Sertoli cells driven by *Amh-Cre* [13]. This model selectively ablates Sertoli cells from the adult testis, causing massive germ cell loss, and has a major effect on the mouse TIF proteome [13]. Sertoli cell-derived proteins would be expected to be decreased in TIF from mice with Sertoli cell ablation. We previously quantified proteins in mouse TIF and showed that 1443 proteins were significantly reduced (p<0.0.05) after Sertoli cell ablation [13]. Our previous study showed that germ cell proteins are contributed to TIF by the seminiferous tubules [13], however the contribution of Sertoli cell-derived proteins to mouse TIF was not investigated. In the current study, we analysed the 1443 proteins in mouse TIF that were significantly reduced by Sertoli cell ablation (Fig 1).

To define which of these 1443 proteins could be contributed to TIF by Sertoli cells, we utilized a previously published RNASeq dataset that allowed us to ascertain which proteins are likely to be predominantly expressed by the Sertoli cells [15]. mRNA expression was measured by RNASeq in whole testes from control adult mice (n = 5) and compared to those with germ cell ablation by the germ cell toxicant busulfan (n = 4), and to mice receiving busulfan with the addition of DTX for 1 week to ablate both germ cells and Sertoli cells (n = 4) [15] (Fig 1). Putative Sertoli cell-derived proteins in TIF were defined by the mRNA transcript corresponding to the down-regulated protein being increased >2 fold in mice with busulfan-induced germ cell ablation compared to controls (suggesting the transcript was enriched in somatic cell mRNA) and being down-regulated >2 fold in mice when Sertoli cells were ablated from busulfan-treated mice (suggesting the transcript was predominantly expressed in Sertoli cells compared to other testicular somatic cells). The thresholds for selection in the RNASeq dataset were chosen to allow a reasonably stringent selection of genes predominantly expressed in Sertoli cells [15]. Whether these genes were actually Sertoli cell-enriched was then further assessed as described below and in Fig 1.

The caveat of the above approach is that the TIF protein may be produced by other testicular somatic cells in a Sertoli cell-dependent manner. For example, Sertoli cell ablation causes alterations in Leydig cell and vascular function and thus could influence gene expression in these cells [4]. Therefore, we next assessed the expression pattern of each of the putative Sertoli cell-derived TIF proteins in a single cell mouse testis RNASeq dataset by Jung and colleagues [14] (Fig 1). In this dataset, Sertoli, Leydig and immune cell-related somatic cell clusters can be defined along with germ cell clusters according to stage of development [14]. We searched each gene symbol in this dataset and examined whether it was predominantly expressed in *Sox9*-expressing cells (Sertoli cells), compared to *Cyp17a1* and *Hsd17b3*-expressing cells (Leydig cells), germ cells and other clusters.

Proteins were then investigated in UniProt, Exocarta (exocarta.org) and PubMed, and examined for possible reproductive phenotypes in the Mouse Genome Informatics (MGI) database (informatics.jax.org/) (Fig 1).

### Identification of Sertoli cell-enriched proteins in human TIF

**Fig 2:**
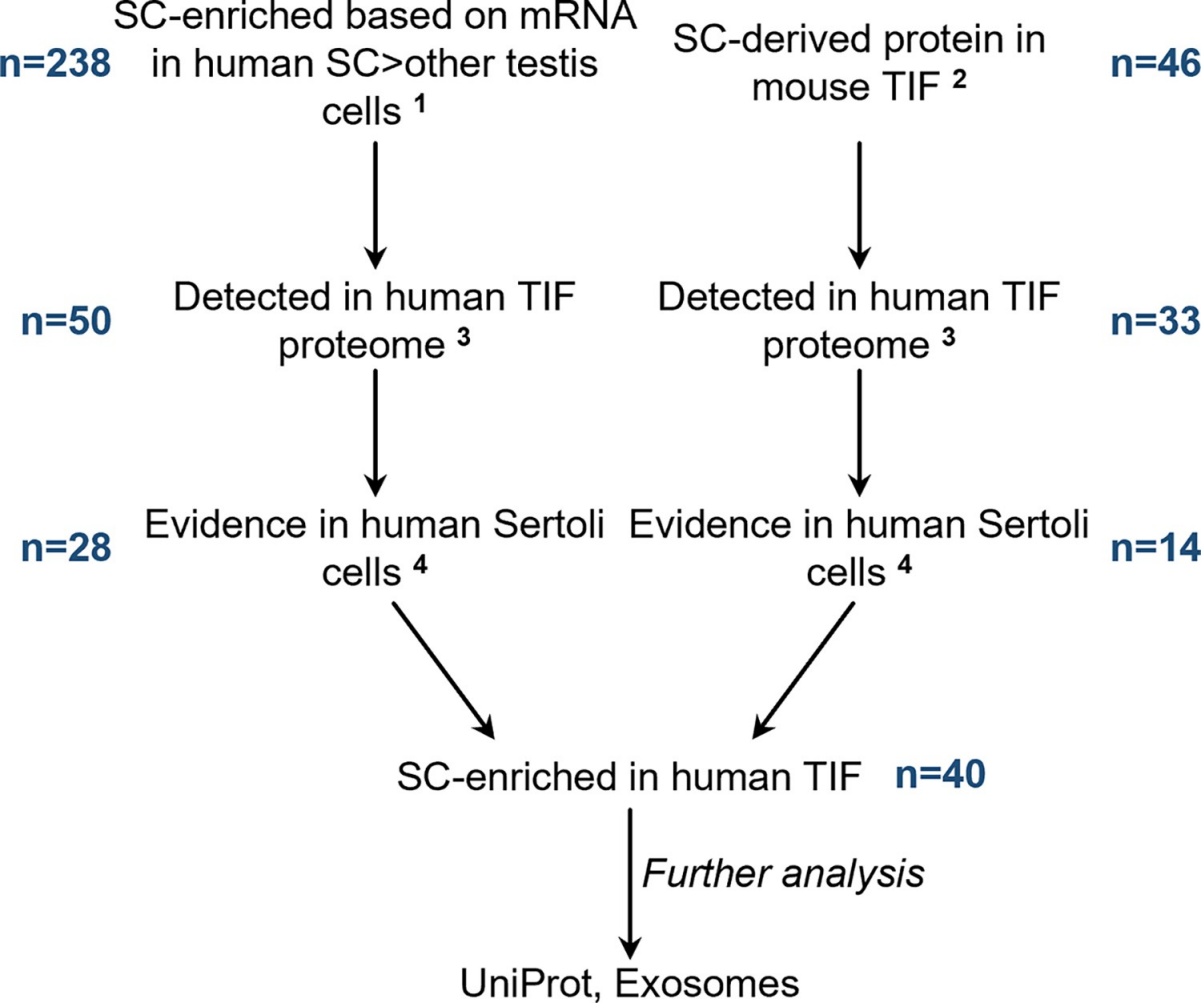
Bioinformatic analysis used to identify Sertoli cell-enriched proteins in human testicular interstitial fluid. *n* indicates the number of proteins that were identified at each step in the analysis. SC = Sertoli cells, ^1^ data on human testis cells downloaded from Human Protein Atlas Tissue Cell tab (https://www.proteinatlas.org/humanproteome/tissue+cell+type/method), ^2^ as identified in Fig 1, ^3^ data from human TIF proteome [13], ^4^ data on mRNA and protein expression and immunolocalization from Human Protein Atlas.

We used two approaches to identify Sertoli cell-enriched proteins in human TIF. First, a dataset of genes that were defined as enriched in Sertoli cells compared to other testis cells was downloaded from Human Protein Atlas [16]. This dataset, from the Tissue Cell tab, consists of n = 238 genes that were deemed to be enriched in human Sertoli cells with an enrichment classification of very high (n = 50) or high (n = 188) compared to other testis cell types based on the human testis cell type-enriched transcriptome (proteinatlas.org/humanproteome/tissue+cell+type/testis). Cell type-enriched genes were subdivided into 3 specificity categories (very high, high or moderate enrichment) as described in the Tissue Cell methods (https://www.proteinatlas.org/humanproteome/tissue+cell+type/method). The Tissue Cell dataset [20] allows an examination of the relative expression of genes in human tissues and cells, including testis somatic cells and germ cells, as distinguished by marker gene expression in an RNASeq dataset on 361 human testes as sourced from the Genotype-Tissue Expression (GTEx) portal (gtexportal.org) via Human Protein Atlas [20]. Note that we did not assess gene expression in the Single Cell section of Human Protein Atlas, because this single cell adult testis RNASeq dataset [21] contained data from only 22 individual Sertoli cells. These putative human Sertoli cell-enriched proteins were assessed for their presence and abundance in human TIF, and for their immunolocalization in human testes in Human Protein Atlas (Fig 2) noting the quality rating of the antibodies used and whether any specific staining indicative of Sertoli cell cytoplasm and/or nucleus was apparent. Proteins were further investigated in UniProt, Exocarta (exocarta.org), and for their expression in mouse testis as described above and in Fig 1.

Second, we investigated the Sertoli cell-enriched proteins that were identified in mouse TIF (Fig 1) and then examined whether the human ortholog of this protein was present in human TIF (Fig 2). If present, the protein and mRNA expression patterns and immunolocalization were examined in Human Protein Atlas datasets as described above and in Fig 2.

### Immunohistochemistry

Testes from phenotypically normal adult mice were fixed in Bouin’s solution for 6 hours, stored in ethanol 70% and embeded in paraffin as described previously [4,22]. Sections of 5 μm were dewaxed in xylene, rehydrated in graded ethanol solutions. For immunohistochemistry, slides were antigen-retrieved in a pressure cooker with 0.01M citrate buffer (pH 6.0). Following inactivation of endogenous peroxidases in 0.3% hydrogen peroxide (v/v) in TBS for 30 min at room temperature, slides were incubated in the appropriate blocking serum for 30 min at room temperature to block the nonspecific activity. The primary antibody was incubated overnight at 4°C diluted in blocking serum. After washing in TBS, slides were incubated for 30 min at room temperature with the appropriate secondary antibody conjugated to biotin diluted 1:500 in the blocking serum. Sections were washed and incubated with horseradish peroxidase-labeled avidin-biotin complex solution (VectorLabs, Peterborough, UK) diluted 1:1000 in TBS for 30 min at room temperature, followed by incubation with the chromogen diaminobenzidine (Immpact DAB; VectorLabs, Peterborough, UK) as per manufacturer’s instructions. Slides were then counterstained with haematoxylin, dehydrated and mounted with Pertex mounting medium (Cell Path, Hemel Hempstead, UK). A Provis microscope (Olympus Optical, London, UK) fitted with a DCS330 digital camera (Eastman Kodak, Rochester, NY) was used to analyse slides.

The primary antibodies used in this study were: 1) EFHD2 rabbit polyclonal (Antibodies online Cat# ABIN1031364 and blocking peptide #ABIN1383010) used at a dilution of 1:200, 2) MARKSL1 rabbit monoclonal (Abcam, catalogue # ab184546) used at 1:800 dilution, 3) SDC4 rabbit polyclonal (LSBio, catalogue # LS-B7740) used at 1:250 dilution. The secondary antibodies used were Goat Anti-Rabbit Biotin Secondary (abcam, # ab6720). The specificity of the immunostaining was assessed using no primary antibody, IgG (Rabbit IgG (abcam, # ab172730) or blocking peptide as negative controls.

## Results

### Identification of Sertoli cell proteins in mouse TIF

To identify proteins of Sertoli cell origin in TIF we first investigated the adult mouse TIF proteome in normal mice vs those with acute Sertoli cell ablation, quantifying 3551 proteins [13]. Of these proteins, 1443 (40.6%) were previously shown to be significantly decreased in abundance (p<0.05) in TIF following Sertoli cell ablation suggesting that they arise from the seminiferous tubules or are dependent on seminiferous tubule function [13]. As previously described, many of these proteins likely arose from germ cells within the seminiferous tubules [13] however it is likely that some are of Sertoli cell origin.

Next, we assessed which of these TIF proteins were likely to be of Sertoli cell origin using an RNASeq dataset from whole testes of mice with germ cell or germ cell and Sertoli cell ablation [15] (see Fig 1 and *Materials and Methods*). Using this approach, we identified 63 putative Sertoli cell-enriched proteins in mouse TIF (Fig 1 and S1A Table in S1 Table). Of these proteins, 31 and 12 were annotated as cytoplasmic or cytoskeletal proteins, respectively (S1A Table in S1 Table).

More detailed analysis of the 63 TIF proteins predicted 46 to be enriched in mouse Sertoli cells compared to other testicular cells based on a single cell RNASeq analysis of mouse testis cells [14] (Fig 1, Table 1 and S1A Table in S1 Table). This list included well-known Sertoli cell-enriched proteins including clusterin (CLU), cathepsin L (CTSL), eppin (EPPIN, encoded by the *Eppin*/*Spinlw1* gene), espin (ESPN) and vinculin (VCL). Clusterin, cathepsin L and eppin are secreted by Sertoli cells [23–25] whereas espin and vinculin are Sertoli cell-enriched intracellular proteins involved in cell adhesion junctions [16].

**Table 1:** Mouse Sertoli cell-enriched proteins in TIF^a^.

Uniprot ID^b^	Gene symbol	Protein name	Detected in exosomes*
B2RWB7	*9630033F20-Rik*	Fructose-2,6-bisphosphatase TIGAR	No
P55264-2	*Adk* *	Adenosine kinase	Yes
Q3UDY1	*Akr1b3*	Aldose reductase	Yes
Q571M4	*Akr1c13*	Aldo-keto reductase family 1 member C13	Yes
Q91XV3	*Basp1*	Brain acid soluble protein 1	Yes
Q543F6	*Cdk5*	Cyclin-dependent-like kinase 5	Yes
Q549A5	*Clu* *	Clusterin	Yes
E9Q1S9	*Cst12*	Cystatin-12	No
Q9Z0H6	*Cst9*	Cystatin-9	No
Q3UWH6	*Ctsl*	Cathepsin L1	Yes
Q9CWS0	*Ddah1* *	N(G),N(G)-dimethylarginine dimethylaminohydrolase 1	Yes
A0A0U1RNK7	*Dock7*	Dedicator of cytokinesis protein 7	Yes
Q8C845	*Efhd2* *	EF-hand domain-containing protein D2	Yes
Q9DA01	*Eppin*	Eppin	No
Q9ET47	*Espn* *	Espin	No
Q8C0D2	*Etnk2*	Ethanolamine kinase 2	No
Q497I3	*Fabp5*	Fatty acid-binding protein, epidermal	Yes
Q8VDH1-2	*Fbxo21* *	F-box only protein 21	No
Q8VC88	*Gca*	Grancalcin	Yes
Q8R5M0	*Gipc3*	PDZ domain-containing protein GIPC3	No
O35660	*Gstm6*	Glutathione S-transferase Mu 6	No
Q80W21	*Gstm7*	Glutathione S-transferase Mu 7	Yes
Q3U4Q5	*Hdac6*	Histone deacetylase 6	Yes
Q9WU63	*Hebp2*	Heme-binding protein 2	Yes
E9Q5B5	*Hk2* *	Hexokinase-2	No
Q9JL35	*Hmgn5* *	High mobility group nucleosome-binding domain-containing protein 5	No
Q545F4	*Hspb1*	Heat shock protein beta-1	Yes
A0A0U1RPG8	*Kctd14*	Potassium channel tetramerisation domain-containing 14	Yes
P16125	*Ldhb*	L-lactate dehydrogenase B chain	Yes
P28667	*Marcksl1* *	MARCKS-related protein 1	Yes
A0A0U1RPX7	*Myo7a*	Unconventional myosin-VIIa	Yes
Q9DCJ9	*Npl*	N-acetylneuraminate lyase	No
B2RQ13	*Pald1*	Paladin	Yes
Q921X9	*Pdia5*	Protein disulfide-isomerase A5	Yes
Q61753	*Phgdh*	D-3-phosphoglycerate dehydrogenase	Yes
Q9WTX2	*Prkra*	Interferon-inducible double-stranded RNA-dependent protein kinase activator A	Yes
Q4VA55	*Pwwp3b*	PWWP domain-containing DNA repair factor 3B	Yes
Q3UFN2	*Qpct*	Glutaminyl-peptide cyclotransferase	Yes
Q4TU85	*Rhox8*	Reproductive homeobox on X chromosome 8	No
A0A158SIT0	*Sat2* *	Diamine acetyltransferase 2	Yes
Q9CZC8	*Scrn1* *	Secernin-1	Yes
Q3UKZ1	*Sdc4* *	Syndecan-4	Yes
Q9DC77	*Smpx*	Small muscular protein	No
A0A0R4J018	*Tpmt* *	Thiopurine S-methyltransferase	Yes
Q9ERD7	*Tubb3*	Tubulin beta-3 chain	Yes
Q64727	*Vcl* *	Vinculin	Yes

^a^ Proteins in mouse TIF were deemed to be enriched in Sertoli cells as described in Fig 1 using data shown in S1A Table in S1 Table.

^b^ Leading UniProt ID identified by mass spectrometry.

^c^ Protein has been detected in exosomes according to Exocarta database (exocarta.org).

* Also detectable in human TIF and show evidence for enrichment in human Sertoli cells, see S1A Table in S1 Table.

These 46 proteins (Table 1) were then further investigated in terms of their likely mechanisms of release from Sertoli cells, their functions, and potential roles in the testis (Fig 1 and S1B Table in S1 Table). Many of these proteins have not yet been shown to have a functional role in the testis (9630033F20RIK/TIGAR, ADK, AKR1C13, CST12, CST9, DOCK7, EFHD2, ETNK2, FBXO21, GIPC3, GSTM6, GSTM7, HEBP2, HMGN5, KCTD14, NPL, PALD1, PDIA5, PRKRA, PWWP3B, QPCT, SMPX and TPMT, see S1B Table in S1 Table). Proteins with annotations or previous data suggesting roles in angiogenesis and/or the function of the vasculature and thus are candidates as Sertoli cell-derived factors involved in the regulation of vascular function [3] include ADK, BASP1, CTSL, DDAH1, CLU, FABP5, HSPB1, MARCKSL1, PALD1, QPCT and SDC4 (S1B Table in S1 Table). Proteins with potential roles in Leydig cells and their steroidogenic function include 9630033F20Rik/TIGAR, AKR1B3, CDK5, DDAH1 and SDC4. Sertoli cell-derived proteins that could potentially regulate interstitial immune cells include AKR1B3, FABP5, FBXO21, GCA, MARCKSL1, PHGDH and PRKRA (S1B Table in S1 Table).

**Fig 3:**
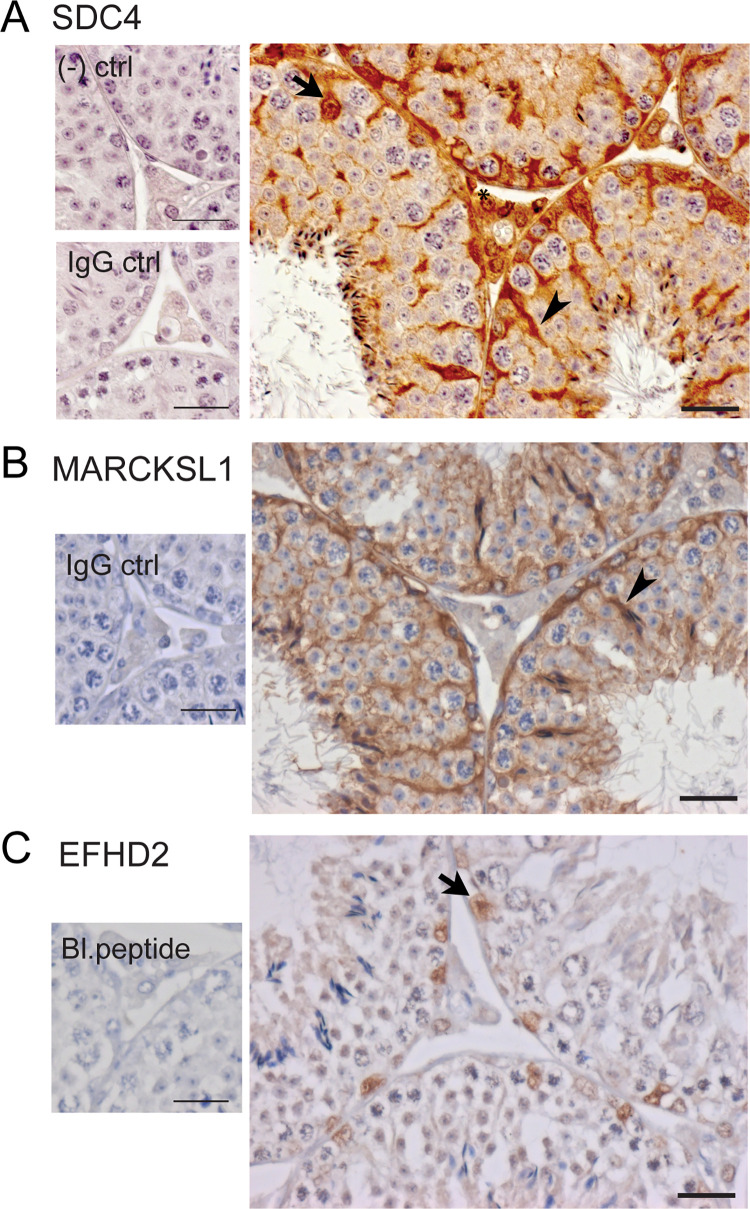
Immunolocalization of putative Sertoli-enriched proteins in adult mouse testis. A. SDC4 was immunolocalized in Sertoli cell cytoplasm (arrowheads) and nuclei (arrows) and in interstitial Leydig cells (asterisk). B. MARCKSL1 was localized in Sertoli cell cytoplasm (arrowheads). C. EFHD2 was localized in Sertoli cell nuclei (arrows). Bar = 25μm. *Abbreviations*: *(-) ctrl*: negative control (primary antibody substituted with buffer); *IgG ctrl*: IgG control (primary antibody substituted with the same concentration of non-immunised IgG); *Bl*.*peptide*: blocking peptide control (primary antibody substituted with blocking peptide for the primary antibody).

In addition to our analyses identifying well-established Sertoli cell proteins, we sought to further verify our approach to selecting Sertoli cell-derived proteins in mouse TIF, through the determination of immunohistochemical localization of 3 proteins, identified in our study, that have not been previously described in Sertoli cells. *Marcksl1*, *Efhd2* and *Sdc4* mRNA are enriched in mouse and human Sertoli cells (Table 1 and S1B Table in S1 Table). EFHD2 was localised to Sertoli cell nuclei whereas MARCKSL1 and SDC4 protein showed a strong, specific localization in Sertoli cell cytoplasm in adult mouse testis (Fig 3). SDC4 was also immunolocalized to interstitial cells, particularly Leydig cells, (Fig 3). We note that single cell RNASeq data suggest this gene is not expressed in adult mouse interstitial cells [14] thus this immunostaining could be non-specific or indicate uptake of the protein by Leydig cells. The demonstration of EFHD2, MARCKSL1 and SDC4 localization to mouse Sertoli cells validates our bioinformatic approach to the identification of Sertoli cell-enriched proteins in mouse TIF.

In summary, using this approach we identified 46 Sertoli cell-enriched proteins that are released into mouse TIF and that could potentially regulate a broad range of functions in the adult testis.

### Identification of Sertoli cell proteins in human TIF

We used two approaches to define potential Sertoli cell-enriched proteins in human TIF (Fig 2). First, we utilized a publicly available dataset on genes that are enriched in human Sertoli cells compared to other testicular cells in Human Protein Atlas (see Fig 2 and *Materials and Methods*). Of the 238 genes that were deemed to be very highly enriched or highly enriched in human Sertoli cells, 49 unique genes had protein products detectable in human TIF and one gene had 2 protein isoforms detectable in TIF (Fig 2 and S2A Table in S2 Table). A further 107 proteins in human TIF were deemed to be moderately enriched in human Sertoli cells compared to other testis cells (S2A Table in S2 Table), however we noted that most of these showed significant expression in other testis cell types so there was insufficient evidence to suggest that they are Sertoli cell-enriched proteins in human TIF.

The proteins that were very highly or highly enriched in human Sertoli cells were further investigated for their immunolocalization in human testis sections using Human Protein Atlas [16] (Fig 2). Of these, 28 showed immunohistochemical localization in Sertoli cells, consistent with the hypothesis that these TIF proteins are enriched in Sertoli cells in the human testis (Table 2 and S2A Table in S2 Table). A further 10 proteins had some immunohistochemical evidence for localization in Sertoli cells but would require further assessment to establish specificity of immunostaining (S2A Table in S2 Table). Of the 28 proteins enriched in Sertoli cells, 3 were also found to be enriched in mouse Sertoli cells; ESPN, HMGN5 and UNC119 (Table 2 and S2A Table in S2 Table). However, 14 of the human TIF proteins enriched in Sertoli cells had no orthologs detected in mouse TIF (S2A Table in S2 Table). In summary, this analysis revealed 28 Sertoli cell-enriched proteins in human TIF. Three of these proteins are also Sertoli cell-enriched TIF proteins in mice.

**Table 2:** Human Sertoli cell-enriched proteins in TIF^a^.

Uniprot ID^b^	Gene symbol	Protein name	Detected in exosomes^c^
H3BRN4	*ABAT*	4-aminobutyrate aminotransferase, mitochondrial	Yes
P33897	*ABCD1*	ATP-binding cassette sub-family D member 1	No
P55263-2	*ADK* *	Adenosine kinase	Yes
P30837	*ALDH1B1*	Aldehyde dehydrogenase X, mitochondrial	Yes
Q14012	*CAMK1*	Calcium/calmodulin-dependent protein kinase type 1	No
P10909-4	*CLU* *	Clusterin	Yes
Q8N1G2	*CMTR1*	Cap-specific mRNA (nucleoside-2-O-)-methyltransferase 1	Yes
P78310-3	*CXADR*	Coxsackievirus and adenovirus receptor	Yes
H7C0R7	*CYB5R1*	NADH-cytochrome b5 reductase 1	Yes
O94760	*DDAH1* *	N(G),N(G)-dimethylarginine dimethylaminohydrolase 1	Yes
Q96C19	*EFHD2* *	EF-hand domain-containing protein D2	Yes
A0A1B0GUN9	*ESPN* *	Espin	No
H0YIE9	*FBXO21* *	F-box only protein 21	No
Q16658	*FSCN1*	Fascin	Yes
P11413	*G6PD*	Glucose-6-phosphate 1-dehydrogenase	Yes
A0A0A0MS68	*GAS7*	Growth arrest-specific protein 7	Yes
H0YKW9	*GATM*	Glycine amidinotransferase, mitochondrial	No
P49448	*GLUD2*	Glutamate dehydrogenase 2, mitochondrial	No
P36959	*GMPR*	GMP reductase 1	Yes
E9PB90	HK2*	Hexokinase-2	No
Q5JSK7	HMGN5*	High mobility group nucleosome-binding domain-containing protein 5	No
C9J8Z4	*IGSF8*	Immunoglobulin superfamily member 8	Yes
P49006	MARCKSL1*	MARCKS-related protein	Yes
Q96N66	*MBOAT7*	Lysophospholipid acyltransferase 7	Yes
Q9NVZ3	*NECAP2*	Adaptin ear-binding coat-associated protein 2	Yes
P17858	*PFKL*	ATP-dependent 6-phosphofructokinase, liver type	Yes
Q9UKY0	*PRND*	Prion-like protein doppel	No
J3KR55	*PTPN7*	Tyrosine-protein phosphatase non-receptor type 7	Yes
Q96F10	SAT2*	Diamine acetyltransferase 2	Yes
Q12765	SCRN1*	Secernin-1	Yes
P31431-2	SDC4*	Syndecan 4	Yes
Q9NQ40-2	*SLC52A3*	Solute carrier family 52 member 3	No
Q01650	*SLC7A5*	Solute carrier family 7 member 5	Yes
P12931	*SRC*	SRC proto-oncogene, non-receptor tyrosine kinase	Yes
O94804	*STK10*	Serine/threonine-protein kinase 10	Yes
P43405-2	*SYK*	Tyrosine-protein kinase SYK	Yes
P51580	TPMT*	Thiopurine S-methyltransferase	Yes
Q13432	UNC119*	Protein unc-119 homolog A	No
P18206-2	VCL*	Vinculin	Yes
A0A0A0MSG0	*WIPF3*	WAS/WASL-interacting protein family member 3	No

^a^ Human TIF proteins were defined as Sertoli cell-enriched according to the criteria shown in Fig 2 and data shown in S2A Table in S2 Table.

^b^ Leading UniProt ID identified by mass spectrometry.

^c^ Protein has been detected in exosomes according to Exocarta database (exocarta.org).

* Also a Sertoli cell-enriched protein in mouse TIF, see S2A Table in S2 Table.

We next investigated whether human orthologs of the 46 Sertoli cell-enriched proteins in mouse TIF were detectable in human TIF (Table 1 and Fig 2). We previously showed that n = 4720 proteins were detectable in TIF from 3 men with normal testis function but had testis biopsies taken for obstructive azoospermia [13]. Of the 46 Sertoli cell-enriched proteins in mouse TIF (Table 1), 33 were also detected in human TIF (Fig 2 and S1A Table in S1 Table). Of these 33 proteins, 14 had mRNA and protein expression patterns consistent with enrichment in human Sertoli cells compared to other testicular cells (Table 1, S1A Table in S1 Table and Fig 2). The three proteins, EFHD2, MARCKSL1 and SDC4 that were verified to be enriched in Sertoli cells in mouse testis by immunohistochemistry (Fig 3) also had evidence to suggest they were enriched in human Sertoli cells and were detectable in human TIF, as were the proteins ADK, CLU, DDAH1, ESPN, FBXO21, HK2, HMGN5, SAT2, SCRN1, TPMT and VCL (Table 1 and S1A Table in S1 Table). Thus, the evidence suggests that these 14 proteins are deposited into TIF by both mouse and human Sertoli cells (Fig 2 and Table 1).

The remaining 19 proteins in human TIF that were orthologues of Sertoli cell-enriched proteins identified in mouse TIF had good evidence for expression in human testis cells (S1A Table in S1 Table). Of these, 10 were expressed in human Sertoli cells but also showed expression in other testis cells, so they could not be classified as Sertoli cell-enriched (S1A Table in S1 Table). The remaining 9 proteins showed evidence of mRNA expression in human germ cells instead of Sertoli cells (S1A Table in S1 Table), suggesting that their cellular sites of expression are not conserved between mouse and human.

In total, we identified a total of 40 Sertoli cell-enriched proteins in human TIF using two different bioinformatic approaches (Fig 2 and Table 2). Of these, 14 were identified by interrogating human datasets for orthologs of Sertoli cell-enriched proteins in mouse TIF (Figs 1 and 2, Table 1 and 2, and S1A Table in S1 Table), 28 proteins were identified by interrogating human testis datasets (Fig 2, Table 2, S2A Table in S2 Table) and 2 (ESPN and HMGN5) were found using both methods.

**Fig 4:**
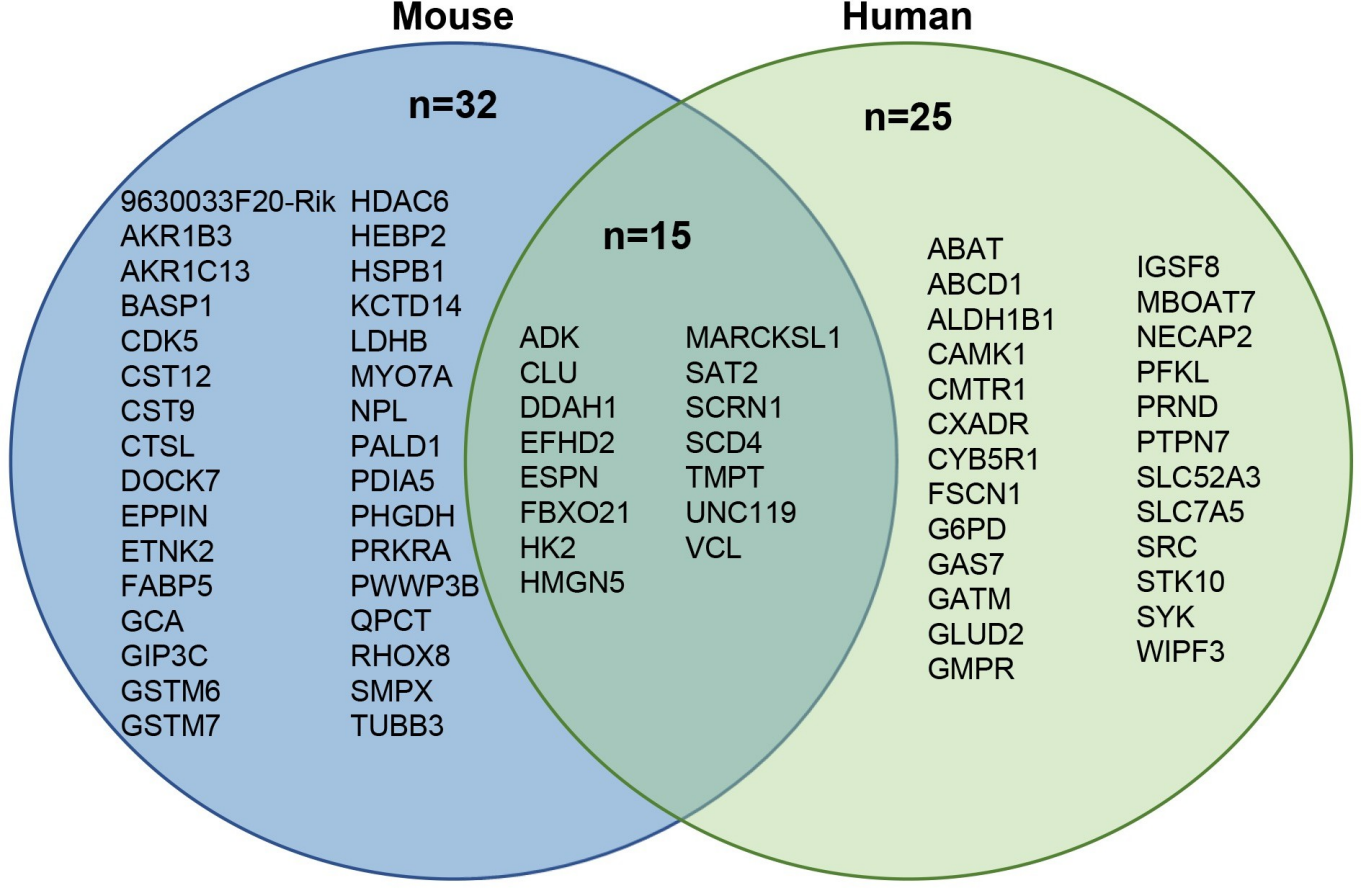
Venn diagram of Sertoli cell-enriched proteins identified in mouse TIF, human TIF or both. Data is shown in S2D Table in S2 Table.

In summary, by interrogating independently generated mouse and human datasets, we identified a total of 47 Sertoli cell-enriched proteins in mouse TIF (Fig 4); 46 of these were identified in mouse TIF (Table 1) and 1 (UNC119) was identified in human TIF but its ortholog was detected in mouse TIF and fulfilled all the criteria for selection as a mouse Sertoli cell-enriched protein (S2A Table in S2 Table). We also identified a total of 40 Sertoli cell-enriched proteins in human TIF (Fig 4). We identified 32 and 25 Sertoli cell-enriched proteins found uniquely in mouse and human TIF, respectively, and 15 proteins in mouse and human TIF that are Sertoli cell-enriched proteins in both species (Fig 4, Tables 1 and 2 and S2D Table in S2 Table).

### Sertoli cell-enriched proteins in TIF could be released via extracellular vesicles

We noted that many of the Sertoli cell-enriched proteins in mouse and human TIF lacked annotations suggesting they are secreted proteins. To understand why these proteins are detectable in TIF from normal mice, but are decreased in mice with Sertoli cell ablation, we examined whether these proteins have been identified in secreted extracellular vesicles (EVs) such as exosomes.

First, we investigated whether exosomal markers proteins were present in mouse TIF. Of the top 100 exosomal markers according to the Exocarta database, we found that 81 were detectable in mouse TIF (S1D Table in S1 Table). Of these, 41 were significantly decreased by Sertoli cell ablation (S1D Table in S1 Table) suggesting that Sertoli cells could deposit exosomal proteins into TIF. Of the 46 Sertoli cell-enriched proteins identified in mouse TIF, 32 have previously been detected in exosomes according to the Exocarta database (Table 1). Furthermore, a previous study identified proteins in extracellular vesicles released by immature porcine Sertoli cells *in vitro* under basal and FSH/testosterone-stimulated conditions [26]. Of the 35 proteins described in porcine Sertoli cell EVs [26], 20 of these were detectable in mouse TIF and 11 were significantly reduced in TIF by acute Sertoli cell ablation (S1C Table in S1 Table).

We also investigated exosomal markers in human TIF and found that 91 of the top 100 exosomal markers according to the Exocarta database were detected (S2C Table in S2 Table). Of the 40 Sertoli cell-enriched proteins in human TIF, 28 have been previously detected in exosomes (Table 2 and S2A Table in S2 Table). In addition, a recent study reported the proteomic composition of EVs released by a human Sertoli cell line *in vitro* and noted that 37 proteins in these EVs were enriched in Sertoli cells in the testis [27,28]. We found that 28 of these proteins were detected in human TIF (S2A and S2B Tables in S2 Table).

Taken together, these data suggest that mouse and human Sertoli cells release EVs, such as exosomes, into TIF and this could be one mechanism by which Sertoli cell-enriched proteins are deposited into TIF.

## Discussion

Sertoli cells are required for normal somatic cell function in the testis [1,6] and thus identifying the factors and mechanisms by which they regulate other testis cells will broaden our understanding of the regulation of optimal testis function. We reasoned that identifying Sertoli cell-enriched proteins in TIF could provide important information as to how these cells communicate with other testicular somatic cells. We interrogated the proteome of mouse TIF and identified proteins that were significantly decreased in TIF when Sertoli cells were ablated from the adult testis [13] and used a bioinformatics approach to define which of these proteins were likely enriched in Sertoli cells based on published RNASeq datasets [14,15]. We also developed a bioinformatic approach to the identification of Sertoli cell-enriched proteins in human TIF proteins, investigating human orthologs of the mouse Sertoli cell-enriched proteins and interrogating a human testis cell type enriched dataset [16]. Using these approaches, we identified 47 and 40 Sertoli cell-enriched proteins in mouse and human TIF, respectively, and 15 proteins that were conserved in both species (Fig 4).

The ability of our bioinformatics approach to identify Sertoli cell-enriched proteins in TIF is supported by the fact that we identified proteins that have previously been shown to be highly enriched in Sertoli cells. Cathepsin L (CTSL) is produced by Sertoli cells in a stage-dependent manner, is responsive to FSH and is secreted into the lumen of the seminiferous tubules [25,29], however its secretion into TIF has not been described. Eppin is encoded by the androgen-responsive gene *Eppin*/*Spinlw1* and the protein is also secreted by Sertoli cells into the seminiferous tubule lumen where it binds to sperm to regulate function and motility [23,30]. Our data suggest that CTSL and EPPIN are bi-directionally secreted by Sertoli cells and could impact on somatic cells as well as germ cells. Clusterin (also known as SGP-2) is a major secretory product of Sertoli cells [24] and our analyses suggest that it is deposited by Sertoli cells into both mouse and human TIF. Espin is another protein that is specifically expressed in the mouse and human testis by Sertoli cells [16,31] and, although it is annotated as an intracellular protein [16], it was detected in mouse and human TIF. Espin is an integral component of a Sertoli cell junctional complex known as the ectoplasmic specialization (ES) [31] which is removed from its association with spermatids via apical tubulobulbar complexes [32]. Since ES-derived proteins can be packaged into cytoplasmic vesicles by the Sertoli cell during sperm release [33,34], it is possible that espin could be released by the seminiferous epithelium in a manner similar to residual body-derived vesicles as previously described [35]. We also identified other known Sertoli cell-enriched proteins in mouse TIF including SCRN1 [36] and vinculin [32]. Therefore, these analyses identified proteins in TIF known to be specifically produced/released by Sertoli cells.

Our approach also identified Sertoli cell-enriched proteins in TIF that have not previously been described in these cells. Immunohistochemical analyses revealed that EFHD2 protein was detected in mouse Sertoli cell nuclei whereas MARCKSL1 and SDC4 were detected in Sertoli cell cytoplasm and nuclei. According to data from the Human Protein Atlas, EFHD2 protein is localized to human Sertoli cell nuclei and perinuclear cytoplasm [16] and is expressed in a range of cell types in the body, where it can regulate calcium signalling, vesicle transport, signal transduction and cellular stress responses [37]. MARCKSL1 is also localized in human Sertoli cell cytoplasm in Human Protein Atlas [16], though its role in the testis has not been described. MARCKSL1 appears to be particularly important in regulating actin filaments that modulate cell structural stability and the function of cytoskeletal protrusions [38,39] suggesting it could contribute to the dynamic changes in Sertoli cell cytoplasmic remodelling during spermatogenesis [40]. However, as a Sertoli cell-enriched protein released into TIF, it could potentially regulate testicular vascular endothelial cell function, given its functional role in angiogenesis [39]. Syndecan-4 (*SDC4*) mRNA is highly enriched in human Sertoli cells however its protein immunolocalization varies between antibodies [16]. SDC4 has been shown to be expressed in pubertal Sertoli cells [41] where it may act as a co-receptor for basic fibroblastic growth factor [42], however its role in adult Sertoli cells or other testis functions has not been described. Other Sertoli cell-enriched TIF proteins of functional interest in the regulation of interstitial cell function include DDAH1 that regulates nitric oxide (NO) generation [43] and could potentially modulate NO-dependent testicular vascular endothelial cell function or Leydig cell steroidogenesis [27,43], and adenosine kinase (ADK) that regulates tissue adenosine levels which are particularly important in regulating vascular function [44]. We identified Sertoli cell-enriched proteins in TIF that have functions consistent with putative roles in regulating Leydig cells (e.g. AKR1B3, CDK5 and DDAH1), resident immune cells (e.g. AKR1B3, FABP5, GCA, MARCKSL1 and PRKRA), and the testicular vasculature (e.g. ADK, BASP1, FABP5, HSPB1 and QPCT), however the function of these proteins in the testis requires further investigation.

There are likely multiple mechanisms by which Sertoli cell-enriched proteins are released into TIF. Sertoli cells could release proteins via secretion or by producing EVs at particular stages. EVs could be released during different stages of spermatogenesis, such as during the mid-spermatogenic stages [13] and after sperm release when proteins in the residual spermatid cytoplasm are packaged into a residual body and can egress from the tubules [35]. We showed that exosomal marker proteins were identified in both mouse and human TIF. We also identified Sertoli cell-enriched proteins in mouse TIF that have been detected in EVs released by pre-pubertal porcine Sertoli cells [26] and 28 human Sertoli cell-enriched TIF proteins that have been detected in EVs released by human Sertoli cells *in vitro* [28]. We propose that Sertoli cells deposit proteins into TIF via secretion and via the release of EVs, including exosomes, and that these vesicles could be one mechanism by which Sertoli cells regulate other somatic cells in the testis [1].

The seminiferous tubules are surrounded by peritubular myoid cells and their associated extracellular matrix, and thus Sertoli cell-enriched proteins would need to be transported across this cellular layer. In mice, the seminiferous tubules are surrounded by a single layer of peritubular myoid cells and their associated basement membrane, whereas in humans, 5–7 layers surround the tubules [45]. The mechanisms by which Sertoli cell proteins and vesicles are transported across these cell layers are unknown. Presumably such transport requires controlled, active processes that may vary between the species, given the multiple layers of cells surrounding human seminiferous tubules. Different mechanisms of protein transport across the peritubular myoid cell layers could partly explain why we identified Sertoli cell-enriched proteins that were unique to mouse and human TIF. Peritubular myoid cells are known to be important for Leydig cell function [46] and perhaps one mechanism by which they regulate testis function could be via the active transport of Sertoli cell-derived vesicles that can then regulate Leydig cell function.

## Conclusions

We have identified proteins that are contributed to TIF by Sertoli cells in mice and humans. Some of these are well known Sertoli cell-enriched proteins whereas others have not previously been described in Sertoli cells or in the testis. Some of these Sertoli cell-enriched proteins in TIF have been detected in EVs, and it is likely that Sertoli cells release EVs at different stages of spermatogenesis. These Sertoli cell-enriched proteins in TIF have the potential to act as paracrine factors that regulate testicular somatic cell function, such as Leydig cells, immune cells, and vascular endothelial cells. Further studies using Sertoli cell-specific targeting strategies would be useful to elucidate the functions of these proteins and reveal new regulatory pathways important for optimal testis function.

## Supporting information

S1 Table Further information on proteins in mouse TIF. *S1A Table*. Potential Sertoli cell-enriched proteins in mouse TIF. *S1B Table*. Further analysis of Sertoli cell-enriched proteins in mouse TIF; putative roles based on the literature. *S1C Table*. Proteins detected in mouse TIF that have previously been detected in porcine Sertoli cell-derived extracellular vesicles. *S1D Table*. Exosomal markers detected in mouse TIF and the effects of Sertoli cell ablation (DTX treatment).
(XLSX)

S2 Table Further information on proteins in human TIF. *S2A Table*. Proteins in human TIF that are very highly or highly enriched in Sertoli cells in the human testis according to Human Protein Atlas. *S2B Table*. Proteins in human TIF that are moderately enriched in Sertoli cells in the human testis according to Human Protein Atlas. *S2C Table*. Exosomal marker proteins detected in human TIF. *S2D Table*. Sertoli cell-enriched proteins that are unique to mouse TIF, human TIF or are conserved in both.
(XLSX)
